# The Bézier variant of Kantorovich type *λ*-Bernstein operators

**DOI:** 10.1186/s13660-018-1688-9

**Published:** 2018-04-18

**Authors:** Qing-Bo Cai

**Affiliations:** School of Mathematics and Computer Science, Quanzhou Normal University, Quanzhou, China

**Keywords:** 41A10, 41A25, 41A36, *λ*-Bernstein operators, Basis functions, Modulus of continuity, Rate of convergence, Absolutely continuous functions

## Abstract

In this paper, we introduce the Bézier variant of Kantorovich type *λ*-Bernstein operators with parameter $\lambda\in[-1,1]$. We establish a global approximation theorem in terms of second order modulus of continuity and a direct approximation theorem by means of the Ditzian–Totik modulus of smoothness. Finally, we combine the Bojanic–Cheng decomposition method with some analysis techniques to derive an asymptotic estimate on the rate of convergence for some absolutely continuous functions.

## Introduction

In 1912, Bernstein [1] proposed the famous polynomials, nowadays called Bernstein polynomials, to prove the Weierstrass approximation theorem as follows:

1 $$\begin{aligned} B_{n}(f;x)=\sum_{k=0}^{n}f \biggl(\frac{k}{n} \biggr)b_{n,k}(x), \end{aligned}$$

where $x\in[0,1]$, $n=1,2,\ldots $ , and Bernstein basis functions $b_{n,k}(x)$ are defined as follows:

2 $$b_{n,k}(x)=\left(\begin{array}{c} n\\ k \end{array}\right)x^{k}{\left(1-x\right)}^{n-k}.$$

Based on this, there are many papers that mention Bernstein type operators, we illustrate some of them [2–13]. In 2010, Ye et al. [14] defined the following new Bernstein bases with shape parameter *λ*:

3 $$\begin{aligned} \textstyle\begin{cases} \widetilde{b}_{n,0}(\lambda;x)=b_{n,0}(x)-\frac{\lambda }{n+1}b_{n+1,1}(x),\\ \widetilde{b}_{n,i}(\lambda;x)=b_{n,i}(x)+\lambda (\frac {n-2i+1}{n^{2}-1}b_{n+1,i}(x)-\frac{n-2i-1}{n^{2}-1}b_{n+1,i+1}(x) )& (1\leq i\leq n-1),\\ \widetilde{b}_{n,n}(\lambda;x)=b_{n,n}(x)-\frac{\lambda}{n+1}b_{n+1,n}(x), \end{cases}\displaystyle \end{aligned}$$

where $b_{n,i}(x)$ ($i=0,1,\ldots,n$) are defined in (2), $x\in [0,1]$, $\lambda\in[-1,1]$. They discussed some important properties of the basis functions and the corresponding curves and tensor product surfaces. It must be pointed out that we have more modeling flexibility when adding the shape parameter *λ*.

Recently, Cai et al. [15] introduced the *λ*-Bernstein operators as follows:

4 $$\begin{aligned} B_{n,\lambda}(f;x)=\sum_{k=0}^{n} \widetilde{b}_{n,k}(\lambda;x)f \biggl(\frac{k}{n} \biggr), \end{aligned}$$

where $\widetilde{b}_{n,k}(\lambda;x)$ ($k=0,1,\ldots,n$) are defined in (3) and $\lambda\in[-1,1]$.

In this paper, we propose the Kantorovich type *λ*-Bernstein operators

5 $$\begin{aligned} K_{n,\lambda}(f;x)=(n+1)\sum_{k=0}^{n} \widetilde{b}_{n,k}(\lambda;x) \int _{\frac{k}{n+1}}^{\frac{k+1}{n+1}}f(t)\,dt, \end{aligned}$$

and the Bézier variant of Kantorovich type *λ*-Bernstein operators

6 $$\begin{aligned} L_{n,\lambda,\alpha}(f;x)=(n+1)\sum_{k=0}^{n}Q_{n,k}^{(\alpha)}( \lambda ;x) \int_{\frac{k}{n+1}}^{\frac{k+1}{n+1}}f(t)\,dt, \end{aligned}$$

where

$$\begin{aligned} Q_{n,k}^{(\alpha)}(\lambda;x)= \bigl[J_{n,k}(\lambda;x) \bigr]^{\alpha }- \bigl[J_{n,k+1}(\lambda;x) \bigr]^{\alpha}, \qquad J_{n,k}(\lambda;x)=\sum_{j=k}^{n} \widetilde{b}_{n,k}(\lambda;x), \end{aligned}$$

$\widetilde{b}_{n,k}(\lambda;x)$ ($k=0,1,\ldots,n$) are defined in (3), $\alpha\geq1$, $x\in[0,1]$, and $\lambda\in[-1,1]$.

Obviously, when $\alpha=1$, $L_{n,\lambda,1}(f;x)$ reduce to Kantorovich type *λ*-Bernstein operators (5); when $\lambda=0$, $L_{n,0,\alpha}(f;x)$ reduce to Bernstein–Kantorovich–Bézier operators defined in [13]; when $\lambda=0$, $\alpha=1$, $L_{n,0,1}(f;x)$ reduce to Bernstein–Kantorovich operators defined in [13].

Let

$$\begin{aligned} P_{n,\lambda,\alpha}(x,t)=(n+1)\sum_{k=0}^{n}Q_{n,k}^{(\alpha)}( \lambda ;x)\chi_{k}(t) \end{aligned}$$

and

$$\begin{aligned} R_{n,\lambda,\alpha}(x,t)= \int_{0}^{t}P_{n,\lambda,\alpha}(x,s)\,ds, \end{aligned}$$

where $\chi_{k}(t)$ is the characteristic function on the interval $[\frac{k}{n+1},\frac{k+1}{n+1} ]$ with respect to $[0,1]$. By the Lebesgue–Stieltjes integral representations, we have

7 $$\begin{aligned} L_{n,\lambda,\alpha}(f;x)= \int_{0}^{1}f(t)P_{n,\lambda,\alpha }(x,t)\,dt= \int_{0}^{1}f(t)\,d_{t}R_{n,\lambda,\alpha}(x,t). \end{aligned}$$

The aims of this paper are to study the rate of convergence of operators $L_{n,\lambda,\alpha}$ for $f\in C_{[0,1]}$ and the asymptotic behavior of $L_{n,\lambda,\alpha}$ for some absolutely continuous functions $f\in\Phi_{\mathrm{DB}}$, where the class of functions of $\Phi_{\mathrm{DB}}$ is defined by

8 $$\begin{aligned} \Phi_{\mathrm{DB}}= \biggl\{ f \Big\vert f(x)-f(0)= \int_{0}^{x}\phi(u)\,du; x\geq0; \phi\mbox{ is bounded on }[0,1] \biggr\} . \end{aligned}$$

For a bounded function *f* on $[0,1]$, the following metric forms were first introduced in [12]:

$$\begin{aligned}& \Omega_{x-}(f;\delta_{1})=\sup_{t\in[x-\delta_{1},x]} \bigl\vert f(t)-f(x) \bigr\vert ; \qquad\Omega_{x+}(f; \delta_{2})=\sup_{t\in[x,x+\delta_{2}]} \bigl\vert f(t)-f(x) \bigr\vert ; \\& \Omega_{x}(f;\mu)=\sup_{t\in[x-x/\mu,x+(1-x)/\mu]} \bigl\vert f(t)-f(x) \bigr\vert , \end{aligned}$$

where $x\in[0,1]$ is fixed, $0\leq\delta_{1}\leq x$, $0\leq\delta_{2}\leq 1-x$, and $\mu\geq1$. For the basic properties of $\Omega_{x-}(f;\delta _{1})$, $\Omega_{x+}(f;\delta_{2})$, and $\Omega_{x}(f;\mu)$, refer to [12].

## Some lemmas

For proving the main results, we need the following lemmas.

### Lemma 2.1

([15])

*Let* $e_{i}=t^{i}$, $i=0,1,2$, *and* $n>1$. *For the* *λ*-*Bernstein operators* $B_{n,\lambda}(f;x)$, *we have*

$$\begin{aligned}& B_{n,\lambda}(e_{0};x)=1; \\& B_{n,\lambda}(e_{1};x)=x+\frac{1-2x+x^{n+1}-(1-x)^{n+1}}{n(n-1)}\lambda; \\& B_{n,\lambda}(e_{2};x)=x^{2}+\frac{x(1-x)}{n}+\lambda \biggl[\frac {2x-4x^{2}+2x^{n+1}}{n(n-1)}+\frac{x^{n+1}+(1-x)^{n+1}-1}{n^{2}(n-1)} \biggr]. \end{aligned}$$

### Lemma 2.2

*Let* $e_{i}=t^{i}$, $i=0,1,2$, *and* $n>1$, *for the Kantorovich type* *λ*-*Bernstein operators* $K_{n,\lambda}(f;x)$, *we have the following equalities*:

9 $$\begin{aligned}& K_{n,\lambda}(e_{0};x)=1; \end{aligned}$$

10 $$\begin{aligned}& K_{n,\lambda}(e_{1};x)=x+\frac{1-2x}{2(n+1)}+\frac {1-2x+x^{n+1}-(1-x)^{n+1}}{n^{2}-1} \lambda; \end{aligned}$$

11 $$\begin{aligned}& K_{n,\lambda}(e_{2};x)=x^{2}+\frac{3nx(2-3x)-3x^{2}+1}{3(n+1)^{2}}+2\lambda \biggl[\frac{ (x-2x^{2}+x^{n+1} )n+x^{n+1}-x}{(n-1)(n+1)^{2}} \biggr]. \end{aligned}$$

### Proof

We can obtain (9) easily by the fact that $\sum_{k=0}^{n}\widetilde{b}_{n,k}(\lambda;x)=1$. Next, by (5) and using Lemma 2.1, we have

$$\begin{aligned} K_{n,\lambda}(e_{1};x) =&(n+1)\sum_{k=0}^{n} \widetilde{b}_{n,k}(\lambda ;x) \int_{\frac{k}{n+1}}^{\frac{k+1}{n+1}}t\,dt \\ =&\sum_{k=0}^{n}\widetilde{b}_{n,k}( \lambda;x)\frac{2k+1}{2(n+1)} \\ =&\frac{n}{n+1}B_{n,\lambda}(e_{1};x)+\frac{1}{2(n+1)} \\ =&x+\frac{1-2x}{2(n+1)}+\frac{1-2x+x^{n+1}-(1-x)^{n+1}}{n^{2}-1}\lambda. \end{aligned}$$

Finally,

$$\begin{aligned} K_{n,\lambda}(e_{2};x) =&(n+1)\sum_{k=0}^{n} \widetilde{b}_{n,k}(\lambda ;x) \int_{\frac{k}{n+1}}^{\frac{k+1}{n+1}}t^{2}\,dt \\ =&\sum_{k=0}^{n}\widetilde{b}_{n,k}( \lambda;x)\frac {3k^{2}+3k+1}{3(n+1)^{2}} \\ =&\frac{n^{2}}{(n+1)^{2}}B_{n,\lambda} (e_{2};x )+\frac {n}{(n+1)^{2}}B_{n,\lambda}(e_{1};x)+ \frac{1}{3(n+1)^{2}} \\ =&x^{2}+\frac{3nx(2-3x)-3x^{2}+1}{3(n+1)^{2}}+2\lambda \biggl[\frac{ (x-2x^{2}+x^{n+1} )n+x^{n+1}-x}{(n-1)(n+1)^{2}} \biggr]. \end{aligned}$$

Lemma 2.2 is proved. □

### Lemma 2.3

*For the Kantorovich type* *λ*-*Bernstein operators* $K_{n,\lambda}(f;x)$ *and* $n>1$, *using Lemma *2.2, *we have*

12 $$ \begin{gathered} K_{n,\lambda}(t-x;x)=\frac{1-2x}{2(n+1)}+\frac {1-2x+x^{n+1}-(1-x)^{n+1}}{n^{2}-1}\lambda, \\ K_{n,\lambda} \bigl((t-x)^{2};x \bigr)=\frac{nx(1-x)}{(n+1)^{2}}+ \frac {1-3x(1-x)}{3(n+1)^{2}}+\frac{2\lambda [x^{n+1}(1-x)+x(1-x)^{n+1} ]}{n^{2}-1} \\ \hphantom{K_{n,\lambda} \bigl((t-x)^{2};x \bigr)=}{}-\frac{4x(1-x)\lambda}{(n+1)^{2}(n-1)} \\ \hphantom{K_{n,\lambda} \bigl((t-x)^{2};x \bigr)}\leq\frac{4}{n+1}. \end{gathered} $$

### Lemma 2.4

*For the Bézier variant of Kantorovich type* *λ*-*Bernstein operators* $L_{n,\lambda,\alpha}(f;x)$ *and* $f\in C_{[0,1]}$ *with the sup*-*norm* $\Vert f \Vert:=\sup_{x\in[0,1]}|f(x)|$, *we have*

$$\begin{aligned} \bigl\Vert L_{n,\lambda,\alpha}(f) \bigr\Vert \leq\alpha \Vert f \Vert . \end{aligned}$$

### Proof

Since, for $\alpha\geq1$, we have

$$\begin{aligned} 0< \bigl[J_{n,k}(\lambda;x)\bigr]^{\alpha}-\bigl[J_{n,k+1}( \lambda;x)\bigr]^{\alpha}\leq \alpha\bigl[J_{n,k}( \lambda;x)-J_{n,k+1}(\lambda;x)\bigr]=\alpha\widetilde {b}_{n,k}( \lambda;x). \end{aligned}$$

Then, from (9) and the definition of $L_{n,\lambda,\alpha }(f;x)$, we have

$$\begin{aligned} \bigl\Vert L_{n,\lambda,\alpha}(f) \bigr\Vert \leq\alpha \bigl\Vert K_{n,\lambda}(f) \bigr\Vert \leq\alpha \Vert f \Vert . \end{aligned}$$

 □

### Lemma 2.5

(i) *For* $0\leq y\leq x<1$, *we have*
  13 $$\begin{aligned} R_{n,\lambda,\alpha}(x,y)= \int_{0}^{y}P_{n,\lambda,\alpha}(x,t)\,dt\leq \frac{4\alpha}{(n+1)(x-y)^{2}}. \end{aligned}$$
(ii) *For* $0< x< z\leq1$, *we have*
  14 $$\begin{aligned} 1-R_{n,\lambda,\alpha}(x,z)= \int_{z}^{1}P_{n,\lambda,\alpha}(x,t)\,dt\leq \frac{4\alpha}{(n+1)(z-x)^{2}}. \end{aligned}$$

### Proof

(i) Using (7) and (12), we have

$$\begin{aligned} R_{n,\lambda,\alpha}(x,y) =& \int_{0}^{y}P_{n,\lambda,\alpha}(x,t)\,dt \\ \leq& \int_{0}^{y} \biggl(\frac{x-t}{x-y} \biggr)^{2}P_{n,\lambda,\alpha }(x,t)\,dt \\ \leq&\frac{1}{(x-y)^{2}} \int_{0}^{1}(t-x)^{2}P_{n,\lambda,\alpha}(x,t)\,dt \\ =&\frac{1}{(x-y)^{2}}L_{n,\lambda,\alpha} \bigl((t-x)^{2};x \bigr) \\ \leq&\frac{\alpha}{(x-y)^{2}}K_{n,\lambda} \bigl((t-x)^{2};x \bigr) \\ \leq&\frac{4\alpha}{(n+1)(x-y)^{2}}. \end{aligned}$$

Similarly, (ii) is proved. □

## Main results

As we know, the space $C_{[0,1]}$ of all continuous functions on $[0,1]$ is a Banach space with sup-norm $\Vert f \Vert:=\sup_{x\in [0,1]}|f(x)|$. Let $f\in C[0,1]$, the Peetre’s *K*-functional is defined by $K_{2}(f;t):=\inf_{g\in C_{[0,1]}^{2}}\{ \Vert f-g \Vert+t \Vert g' \Vert+{t}^{2} \Vert g'' \Vert\}$, where $t>0$ and $C_{[0,1]}^{2}:=\{g\in C_{[0,1]}: g', g''\in C_{[0,1]}\}$. By [16], there exists an absolute constant $C>0$ such that

15 $$ K_{2}(f;t)\leq C\omega_{2} (f;\sqrt{t} ), $$

where $\omega_{2}(f;t):=\sup_{0< h\leq t}\sup_{x,x+h,x+2h\in [0,1]}|f(x+2h)-2f(x+h)+f(x)|$ is the second order modulus of smoothness of $f\in C_{[0,1]}$. We also denote the usual modulus of continuity of $f\in C_{[0,1]}$ by $\omega(f;t):=\sup_{0< h\leq t}\sup_{x,x+h\in [0,1]}|f(x+h)-f(x)|$.

### Theorem 3.1

*For* $f\in C_{[0,1]}$, $\lambda\in[-1,1]$, *we have*

16 $$\begin{aligned} \bigl\vert L_{n,\lambda,\alpha}(f;x)-f(x) \bigr\vert \leq C\omega _{2} \biggl(f;\sqrt{\frac{\alpha}{n+1}} \biggr), \end{aligned}$$

*where* *C* *is a positive constant*.

### Proof

Let $g\in C_{[0,1]}^{2}$, by Taylor’s expansion

$$\begin{aligned} g(t)=g(x)+g'(x) (t-x)+ \int_{x}^{t}(t-u)g''(u)\,du. \end{aligned}$$

As we know, $L_{n,\lambda,\alpha}(1;x)=1$. Applying $L_{n,\lambda,\alpha }(\cdot;x)$ to both sides of the above equation, we get

$$\begin{aligned} L_{n,\lambda,\alpha}(g;x)=g(x)+g'(x)L_{n,\lambda,\alpha }(t-x;x)+L_{n,\lambda,\alpha} \biggl( \int_{x}^{t}(t-u)g''(u)\,du;x \biggr). \end{aligned}$$

By the Cauchy–Schwarz inequality, (12) and Lemma 2.4, we have

$$ \begin{aligned} \bigl\vert L_{n,\lambda,\alpha}(g;x)-g(x) \bigr\vert &\leq \bigl\vert g'(x) \bigr\vert \bigl\vert L_{n,\lambda,\alpha}\bigl( \vert t-x \vert ;x\bigr) \bigr\vert + \biggl\vert L_{n,\lambda,\alpha} \biggl( \int _{x}^{t}(t-u)g''(u)\,du;x \biggr) \biggr\vert \\ &\leq \bigl\Vert g' \bigr\Vert L_{n,\lambda,\alpha}\bigl( \vert t-x \vert ;x\bigr)+\frac{ \Vert g'' \Vert }{2}L_{n,\lambda ,\alpha} \bigl((t-x)^{2};x \bigr) \\ &\leq \bigl\Vert g' \bigr\Vert \sqrt{L_{n,\lambda,\alpha} \bigl((t-x)^{2};x \bigr)}+\frac{ \Vert g'' \Vert }{2}L_{n,\lambda ,\alpha} \bigl((t-x)^{2};x \bigr) \\ &\leq\sqrt{\alpha} \bigl\Vert g' \bigr\Vert \sqrt{K_{n,\lambda} \bigl((t-x)^{2};x \bigr)}+\frac{\alpha \Vert g'' \Vert }{2}K_{n,\lambda} \bigl((t-x)^{2};x \bigr) \\ &\leq\frac{2\sqrt{\alpha} \Vert g' \Vert }{\sqrt{n+1}}+\frac {2\alpha \Vert g'' \Vert }{n+1}. \end{aligned} $$

Then, using the above inequality, we have

$$\begin{aligned} \bigl\vert L_{n,\lambda,\alpha}(f;x)-f(x) \bigr\vert \leq& \bigl\vert L_{n,\lambda,\alpha}(f-g;x) \bigr\vert + \bigl\vert (f-g) (x) \bigr\vert + \bigl\vert L_{n,\lambda,\alpha}(g;x)-g(x) \bigr\vert \\ \leq&2 \biggl( \Vert f-g \Vert +\sqrt{\frac{\alpha}{n+1}} \bigl\Vert g' \bigr\Vert +\frac{\alpha}{n+1} \bigl\Vert g' \bigr\Vert \biggr). \end{aligned}$$

Hence, taking infimum on the right-hand side over all $g\in C_{[0,1]}^{2}$, we get

$$\begin{aligned} \bigl\vert L_{n,\lambda,\alpha}(f;x)-f(x) \bigr\vert \leq2K_{2} \biggl(f;\frac{\alpha}{n+1} \biggr). \end{aligned}$$

By (15), we obtain

$$\begin{aligned} \bigl\vert L_{n,\lambda,\alpha}(f;x)-f(x) \bigr\vert \leq C\omega _{2} \biggl(f;\sqrt{\frac{\alpha}{n+1}} \biggr). \end{aligned}$$

This completes the proof of Theorem 3.1. □

Next, we recall some definitions of the Ditzian–Totik first order modulus of smoothness and *K*-functional, which can be found in [17]. Let $f\in C_{[0,1]}$, and $\varphi(x):=\sqrt{x(1-x)}$, the first order modulus of smoothness is given by

$$\begin{aligned} \omega_{\varphi}(f;t):=\sup_{0< h\leq t, x\pm\frac{h\varphi(x)}{2}\in [0,1]} \biggl\vert f \biggl(x+\frac{h\varphi(x)}{2} \biggr)-f \biggl(x-\frac {h\varphi(x)}{2} \biggr) \biggr\vert . \end{aligned}$$

The *K*-functional $K_{\varphi}(f;t)$ is defined by $K_{\varphi}(f;t):=\inf_{g\in C^{\varphi}_{[0,1]}} \{ \Vert f-g \Vert+t \Vert\varphi g' \Vert \}$, where $t>0$, $C^{\varphi}_{[0,1]}:= \{g:g\in AC_{[0,1]}, \Vert \varphi g' \Vert<\infty \}$, $AC_{[0,1]}$ is the class of all absolutely continuous functions on $[0,1]$. Besides, from [17], there exists a constant $C>0$ such that

17 $$\begin{aligned} K_{\varphi}(f;t)\leq C\omega_{\varphi}(f;t). \end{aligned}$$

### Theorem 3.2

*For* $f\in C_{[0,1]}$, $\lambda\in[-1,1]$, *and* $\varphi (x)=\sqrt{x(1-x)}$, *we have*

$$\begin{aligned} \bigl\vert L_{n,\lambda,\alpha}(f;x)-f(x) \bigr\vert \leq C\omega _{\varphi} \biggl(f;\frac{2\sqrt{2\alpha}}{\sqrt{n+1}\varphi(x)} \biggr), \end{aligned}$$

*where* *C* *is a positive constant*.

### Proof

Since

$$\begin{aligned} g(t)=g(x)+ \int_{x}^{t}g'(u)\,du, \end{aligned}$$

applying $L_{n,\lambda,\alpha}(f;x)$ to the above equality, we have

18 $$\begin{aligned} L_{n,\lambda,\alpha}(g;x)=g(x)+L_{n,\lambda,\alpha} \biggl( \int _{x}^{t}g'(u)\,du;x \biggr). \end{aligned}$$

We will estimate $\int_{x}^{t}g'(u)\,du$: For any $x,t\in(0,1)$, we have

$$\begin{aligned} \biggl\vert \int_{x}^{t}g'(u)\,du \biggr\vert \leq& \bigl\Vert \varphi g' \bigr\Vert \biggl\vert \int_{x}^{t}\frac{1}{\varphi(u)}\,du \biggr\vert \\ =& \bigl\Vert \varphi g' \bigr\Vert \biggl\vert \int_{x}^{t}\frac {1}{\sqrt{u(1-u)}}\,du \biggr\vert \\ \leq& \bigl\Vert \varphi g' \bigr\Vert \biggl\vert \int_{x}^{t} \biggl(\frac{1}{\sqrt{u}}+ \frac{1}{\sqrt{1-u}} \biggr)\,du \biggr\vert \\ \leq&2 \bigl\Vert \varphi g' \bigr\Vert \bigl( \vert \sqrt {t}-\sqrt{x} \vert + \vert \sqrt{1-t}-\sqrt{1-x} \vert \bigr) \\ =&2 \bigl\Vert \varphi g' \bigr\Vert \vert t-x \vert \biggl(\frac{1}{\sqrt{t}+\sqrt{x}}+\frac{1}{\sqrt{1-t}+\sqrt{1-x}} \biggr) \\ \leq&2 \bigl\Vert \varphi g' \bigr\Vert \vert t-x \vert \biggl(\frac{1}{\sqrt{x}}+\frac{1}{\sqrt{1-x}} \biggr) \\ \leq&2\sqrt{2} \bigl\Vert \varphi g' \bigr\Vert \frac{ \vert t-x \vert }{\varphi(x)}. \end{aligned}$$

From (18), using the Cauchy–Schwarz inequality, we obtain

$$\begin{aligned} \bigl\vert L_{n,\lambda,\alpha}(g;x)-g(x) \bigr\vert \leq&2\sqrt {2} \frac{ \Vert \varphi g' \Vert }{\varphi(x)}L_{n,\lambda ,\alpha}\bigl( \vert t-x \vert ;x\bigr) \\ \leq&2\sqrt{2}\frac{ \Vert \varphi g' \Vert }{\varphi (x)}\sqrt{L_{n,\lambda,\alpha} \bigl((t-x)^{2};x \bigr)} \\ \leq&2\sqrt{2\alpha}\frac{ \Vert \varphi g' \Vert }{\varphi (x)}\sqrt{K_{n,\lambda} \bigl((t-x)^{2};x \bigr)} \\ \leq&\frac{4\sqrt{2\alpha} \Vert \varphi g' \Vert }{\sqrt {n+1}\varphi(x)}. \end{aligned}$$

Hence, using the above inequality, we have

$$\begin{aligned} \bigl\vert L_{n,\lambda,\alpha}(f;x)-f(x) \bigr\vert \leq& \bigl\vert L_{n,\lambda,\alpha}(f-g;x) \bigr\vert + \bigl\vert (f-g) (x) \bigr\vert + \bigl\vert L_{n,\lambda,\alpha}(f;x)-g(x) \bigr\vert \\ \leq&2 \biggl( \Vert f-g \Vert +\frac{2\sqrt{2\alpha}}{\sqrt {n+1}\varphi(x)} \bigl\Vert \varphi g' \bigr\Vert \biggr). \end{aligned}$$

Taking infimum on the right-hand side over all $g\in C_{[0,1]}^{\varphi }$, we get

$$\begin{aligned} \bigl\vert L_{n,\lambda,\alpha}(f;x)-f(x) \bigr\vert \leq&2K_{\varphi } \biggl(f;\frac{2\sqrt{2\alpha}}{\sqrt{n+1}\varphi(x)} \biggr). \end{aligned}$$

By (17), we obtain

$$\begin{aligned} \bigl\vert L_{n,\lambda,\alpha}(f;x)-f(x) \bigr\vert \leq&C\omega _{\varphi} \biggl(f;\frac{2\sqrt{2\alpha}}{\sqrt{n+1}\varphi(x)} \biggr). \end{aligned}$$

Theorem 3.2 is proved. □

Finally, we study the approximation properties of $L_{n,\lambda,\alpha }(f;x)$ for some absolutely continuous functions $f\in\Phi_{\mathrm{DB}}$.

### Theorem 3.3

*Let* *f* *be a function in* $\Phi_{\mathrm{DB}}$. *If* $\phi(x+)$ *and* $\phi (x-)$ *exist at a fixed point* $x\in(0,1)$, *then we have*

$$\begin{aligned} \bigl\vert L_{n,\lambda,\alpha}(f;x)-f(x) \bigr\vert \leq\frac{2\alpha ( \vert \phi(x+) \vert + \vert \phi(x-) \vert )}{\sqrt{n+1}}+ \frac{8\alpha+2x(1-x)}{nx(1-x)}\sum_{k=1}^{[\sqrt{n}]} \Omega_{x} \biggl(\phi_{x};\frac{1}{k} \biggr), \end{aligned}$$

*where* $[n]$ *denotes the greatest integer not exceeding* *n*, *and*

19 $$\begin{aligned} \phi_{x}(u)= \textstyle\begin{cases} \phi(u)-\phi(x+),&x< u\leq1;\\ 0,&u=x;\\ \phi(u)-\phi(x-),&0\leq u< x. \end{cases}\displaystyle \end{aligned}$$

### Proof

By the fact that $L_{n,\lambda,\alpha}(1;x)=1$, using (7) and (8), we have

$$\begin{aligned} L_{n,\lambda,\alpha}(f;x)-f(x) =& \int_{0}^{1} \bigl[f(t)-f(x) \bigr]\,d_{t}R_{n,\lambda,\alpha}(x,t) \\ =& \int_{0}^{1} \biggl( \int_{x}^{t}\phi(u)\,du \biggr)\,d_{t}R_{n,\lambda,\alpha}(x,t). \end{aligned}$$

By the Bojanic–Cheng decomposition [18], we have

20 $$\begin{aligned} \phi(u) =&\frac{\phi(x+)+\phi(x-)}{2}+\phi_{x}(u)+\frac{\phi(x+)-\phi (x-)}{2}\operatorname{sgn}(u-x) \\ &{}+\delta_{x}(u) \biggl(\phi(x)-\frac{\phi(x+)+\phi(x-)}{2} \biggr), \end{aligned}$$

where $\phi_{x}(u)$ is defined in (19), $\operatorname{sgn}(u)$ is a sign function and $\delta_{x}(u)= \bigl\{ \scriptsize{ \begin{array}{l@{\quad}l} 1,&u=x;\\ 0,&u\neq x. \end{array} } $ By direct integrations, we find that

21 $$\begin{aligned} L_{n,\lambda,\alpha}(f;x)-f(x) =&\frac{\phi(x+)-\phi(x-)}{2}L_{n,\lambda ,\alpha}\bigl( \vert t-x \vert ;x\bigr)-U_{n,\lambda,\alpha}(\phi _{x};x)+T_{n,\lambda,\alpha}( \phi_{x};x) \\ &{}+\frac{\phi(x+)+\phi(x-)}{2}L_{n,\lambda,\alpha}(t-x;x), \end{aligned}$$

where

$$\begin{gathered} U_{n,\lambda,\alpha}(\phi_{x};x)= \int_{0}^{x} \biggl( \int_{t}^{x}\phi _{x}(u)\,du \biggr)\,d_{t}R_{n,\lambda,\alpha}(x,t), \\ T_{n,\lambda,\alpha}(\phi_{x};x)= \int_{x}^{1} \biggl( \int_{x}^{t}\phi _{x}(u)\,du \biggr)\,d_{t}R_{n,\lambda,\alpha}(x,t). \end{gathered} $$

Integration by parts derives

$$\begin{aligned} U_{n,\lambda,\alpha}(\phi_{x};x) =& \int_{0}^{x} \biggl( \int_{t}^{x}\phi _{x}(u)\,du \biggr)\,d_{t}R_{n,\lambda,\alpha}(x,t) \\ =& \int_{t}^{x}\phi_{x}(u)\,duR_{n,\lambda,\alpha}(x,t) \bigg\vert _{0}^{x}+ \int_{0}^{x}R_{n,\lambda,\alpha}(x,t) \phi_{x}(t)\,dt \\ =& \int_{0}^{x}R_{n,\lambda,\alpha}(x,t) \phi_{x}(t)\,dt \\ =& \biggl( \int_{0}^{x-x/\sqrt{n}}+ \int_{x-x/\sqrt{n}}^{x} \biggr)R_{n,\lambda,\alpha}(x,t) \phi_{x}(t)\,dt. \end{aligned}$$

Note that $R_{n,\lambda,\alpha}(x,t)\leq1$ and $\phi_{x}(x)=0$, it follows that

$$\begin{aligned} \biggl\vert \int_{x-x/\sqrt{n}}^{x}R_{n,\lambda,\alpha}(x,t)\phi _{x}(t)\,dt \biggr\vert \leq\frac{x}{\sqrt{n}}\Omega_{x} \biggl(\phi_{x};\frac {x}{\sqrt{n}} \biggr)\leq\frac{2x}{n}\sum _{k=1}^{[\sqrt{n}]}\Omega _{x} \biggl( \phi_{x};\frac{x}{k} \biggr). \end{aligned}$$

From Lemma 2.5 (i) and change of variable $t=x-x/u$, we have

$$\begin{aligned} \biggl\vert \int_{0}^{x-x/\sqrt{n}}R_{n,\lambda,\alpha}(x,t)\phi _{x}(t)\,dt \biggr\vert \leq&\frac{4\alpha}{n+1} \int_{0}^{x-x/\sqrt{n}}\frac {\Omega_{x}(\phi_{x},x-t)}{(x-t)^{2}}\,dt \\ =&\frac{4\alpha}{(n+1)x} \int_{1}^{\sqrt{n}}\Omega_{x} \biggl( \phi_{x};\frac {x}{u} \biggr)\,du \\ \leq&\frac{8\alpha}{(n+1)x}\sum_{k=1}^{[\sqrt{n}]} \Omega_{x} \biggl(\phi _{x};\frac{x}{k} \biggr). \end{aligned}$$

Thus, it follows that

22 $$\begin{aligned} \bigl\vert U_{n,\lambda,\alpha}(\phi_{x};x) \bigr\vert \leq& \frac{8\alpha }{(n+1)x}\sum_{k=1}^{[\sqrt{n}]} \Omega_{x} \biggl(\phi_{x};\frac{x}{k} \biggr)+ \frac{2x}{n}\sum_{k=1}^{[\sqrt{n}]} \Omega_{x} \biggl(\phi_{x};\frac {x}{k} \biggr) \\ \leq&\frac{8\alpha+2x^{2}}{nx}\sum_{k=1}^{[\sqrt{n}]} \Omega_{x} \biggl(\phi _{x};\frac{1}{k} \biggr). \end{aligned}$$

From Lemma 2.5(ii), using a similar method, we also obtain

23 $$\begin{aligned} \bigl\vert T_{n,\lambda,\alpha}(\phi_{x};x) \bigr\vert \leq \frac{8\alpha +2(1-x)^{2}}{n(1-x)}\sum_{k=1}^{[\sqrt{n}]} \Omega_{x} \biggl(\phi_{x};\frac {1}{k} \biggr). \end{aligned}$$

By the Cauchy–Schwarz inequality, (12), and Lemma 2.4, we have

24 $$\begin{aligned} L_{n,\lambda,\alpha}\bigl( \vert t-x \vert ;x\bigr)\leq\alpha K_{n,\lambda} \bigl( \vert t-x \vert ;x\bigr)\leq\alpha\sqrt {K_{n,\lambda} \bigl((t-x)^{2};x \bigr)}\leq\frac{2\alpha}{\sqrt {n+1}}. \end{aligned}$$

Hence, by (22), (23), (24), and (21), we have

$$\begin{aligned} \bigl\vert L_{n,\lambda,\alpha}(f;x)-f(x) \bigr\vert \leq\frac{2\alpha ( \vert \phi(x+) \vert + \vert \phi(x-) \vert )}{\sqrt{n+1}}+ \frac{8\alpha+2x(1-x)}{nx(1-x)}\sum_{k=1}^{[\sqrt{n}]} \Omega_{x} \biggl(\phi_{x};\frac{1}{k} \biggr). \end{aligned}$$

Theorem 3.3 is proved. □

## Conclusion

In this paper, we have presented a Bézier variant of Kantorovich type *λ*-Bernstein operators $L_{n,\lambda,\alpha}(f;x)$, and established approximation theorems by using the usual second order modulus of smoothness and the Ditzian–Totik modulus of smoothness. From Theorem 3.3 of Sect. 3, we know that the rate of convergence of operators $L_{n,\lambda,\alpha}(f;x)$ for $f\in\Phi_{\mathrm{DB}}$ is $\frac {1}{\sqrt{n+1}}$. Furthermore, we might consider the approximation of these operators $L_{n,\lambda,\alpha}(f;x)$ for locally bounded functions.

## References

[1] Bernstein S.N. (1912). Démonstration du théorème de Weierstrass fondée sur la calcul des probabilités. Comm. Soc. Math. Charkow Sér. 2 t..

[2] Agrawal P.N., Gupta V., Kumar A.S. (2013). On *q*-analogue of Bernstein–Schurer–Stancu operators. Appl. Math. Comput..

[3] Ditzian Z., Ivanov K. (1989). Bernstein-type operators and their derivatives. J. Approx. Theory.

[4] Guo S., Li C., Liu X., Song Z. (2000). Pointwise approximation for linear combinations of Bernstein operators. J. Approx. Theory.

[5] Gupta V. (2008). Some approximation properties of *q*-Durrmeyer operators. Appl. Math. Comput..

[6] Mursaleen M., Ansari K.J., Khan A. (2015). On $(p, q)$-analogue of Bernstein operators. Appl. Math. Comput..

[7] Mursaleen M., Ansari K.J., Khan A. (2015). Some approximation results by $(p, q)$-analogue of Bernstein–Stancu operators. Appl. Math. Comput..

[8] Mursaleen M., Ansari K.J., Khan A. (2017). Approximation by Kantorovich type *q*-Bernstein–Stancu operators. Complex Anal. Oper. Theory.

[9] Mursaleen M., Ansari K.J., Khan A. (2016). Some approximation results for Bernstein–Kantorovich operators based on $(p, q)$-calculus. UPB Sci. Bull., Ser. A.

[10] Nowak G. (2009). Approximation properties for generalized *q*-Bernstein polynomials. J. Math. Anal. Appl..

[11] Phillips G.M. (1997). Bernstein polynomials based on the *q*-integers. Ann. Numer. Math..

[12] Zeng X.M., Cheng F. (2001). On the rates of approximation of Bernstein type operators. J. Approx. Theory.

[13] Zeng X.M., Piriou A. (1998). On the rate of convergence of two Bernstein–Bézier type operators for bounded variation functions. J. Approx. Theory.

[14] Ye Z., Long X., Zeng X.M. (2010). Adjustment algorithms for Bézier curve and surface. International Conference on Computer Science and Education.

[15] Cai Q.B., Lian B.Y., Zhou G. (2018). Approximation properties of *λ*-Bernstein operators. J. Inequal. Appl..

[16] DeVore R.A., Lorentz G.G. (1993). Constructive Approximation.

[17] Ditzian Z., Totik V. (1987). Moduli of Smoothness.

[18] Bojanic R., Cheng F. (1989). Rate of convergence of Bernstein polynomials for functions with derivatives of bounded variation. J. Math. Anal. Appl..

